# Case Report: Management of intracranial hypertension in acute-on-chronic liver failure: a case of fulminant cerebral edema and acute-onset severe hyperammonemia in a patient with cirrhosis

**DOI:** 10.3389/fgstr.2025.1542588

**Published:** 2025-03-06

**Authors:** Wei Tang, Makeda Dawkins, Anila Kumar, Mohammed Nasereldin, Gabriel Heering, Morgan Soffler, David C. Wolf

**Affiliations:** ^1^ Department of Internal Medicine, Westchester Medical Center, Valhalla, NY, United States; ^2^ Department of Gastroenterology, Westchester Medical Center, Valhalla, NY, United States; ^3^ Department of Pulmonary and Critical Care Medicine, Westchester Medical Center, Valhalla, NY, United States; ^4^ Department of Transplant Hepatology, Westchester Medical Center, Valhalla, NY, United States

**Keywords:** hyperammonemia, hepatic encephalopathy, acute on chronic liver failure, cerebral edema, cirrhosis, intracranial hypertension (ICH)

## Abstract

Intracranial hypertension (ICH) is a well-recognized and potentially fatal complication of acute liver failure. It is rarely observed in patients with chronic liver disease or acute-on-chronic liver failure (ACLF). Only a few studies have investigated the management of ICH in ACLF. Here, we present an uncommon case of acute-onset severe hyperammonemia in a patient with cirrhosis who developed fulminant ICH. Rapid institution of renal placement therapy and therapeutic plasma exchange achieved a dramatic reduction in the serum ammonia level, but did not slow the patient’s rapid neurological deterioration.

## Introduction

Hyperammonemic cerebral edema and subsequent intracranial hypertension (ICH) are potentially fatal complications of acute liver failure (ALF). Still, they are rarely seen in patients with chronic liver disease (CLD) or acute-on-chronic liver failure (ACLF) (1). Although ammonia plays a role in the development of cerebral edema and ICH, the utility of serial ammonia measurements in hepatic encephalopathy (HE) management is widely debated (2). A variety of therapeutic modalities available for the management of ICH in ALF have been utilized in patients with ACLF. These include: osmotic agents to decrease intracerebral pressure (ICP), permissive hypocapnia, moderate hypothermia, and ammonia-reduction therapies. The latter includes continuous renal replacement therapy (CRRT) and therapeutic plasma exchange (TPE) (3). Here, we present a case of acute-onset severe hyperammonemia and subsequent ICH in a patient with ACLF who failed intensive ICH management.

## Case presentation

A 53-year-old man with a history of decompensated alcohol-associated cirrhosis and end-stage renal disease was admitted with severe nausea and non-bloody emesis for two days. He had a history of refractory ascites, spontaneous bacterial peritonitis and esophageal variceal bleeding. These issues were successfully addressed by a transjugular intrahepatic portosystemic shunt procedure five years prior to admission. Notably, the patient had a history of HE both prior to and after the TIPS procedure. There was no family history of urea cycle disorders. The patient was listed for simultaneous liver-kidney transplant with a Model for End-Stage Liver Disease (MELD) score of 25.

Upon admission, heart rate was 98, blood pressure was 121/83, and the oxygen saturation was 97% on room air. Physical examination was only notable for right lower quadrant tenderness. The patient had normal mental function. Labs on admission (**Table 1**) were notable for stable liver and kidney dysfunction with total bilirubin 4.6 mg/dL, INR 1.52, and creatinine 6.3 mg/dL. The serum lactate was elevated at 5.9 mmol/L.The serum ammonia level was normal at 40 μmol/L. His calculated MELD-Na score was 29. Twelve hours after admission, the patient suddenly became unresponsive to pain. He had a right gaze preference and right lower extremity weakness, which prompted evaluation to rule out stroke. Computed tomography (CT) of the head revealed subtle loss of grey-white matter differentiation. CT angiogram of the head and neck with perfusion analysis was unremarkable. He deteriorated rapidly and developed acute hypoxic respiratory failure that necessitated intubation. Repeat labs (**Table 1**) were grossly unchanged except for a strikingly elevated ammonia level (810 μmol/L), rising serum lactate (10.9 mmol/L), and an elevated procalcitonin (89.4 ng/mL). Abdominal ultrasound with Doppler showed a patent TIPS.

**Table 1:** Laboratory data.

Variable	Reference Range	On admission	6 Hours
Complete Blood Count			
White-cell count (k/mm^3^)	4.80-10.80	9.26	9.11
Hemoglobin (g/dL)	14.0-18.0	16.2	16.5
Platelet (k/mm^3^)	160-410	71	122
Routine Chemistry			
Sodium (mEq/L)	135-145	143	143
Potassium (mEq/L)	3.5-5.1	3.8	4.2
Bicarbonate (mEq/L)	22-30	16	18
BUN (mg/dL)	6-22	68	91
Creatinine	0.72-1.25	6.29	6.48
Hepatic Function Panel			
Albumin	3.4-4.8	4.0	3.3
Total Bilirubin (mg/dL)	0.2-1.3	4.6	3.4
Alkaline phosphatase (U/L)	40-150	165	118
AST (U/L)	4-35	14	14
ALT (U/L)	6-55	11	8
Coagulation Studies			
INR	0.90-1.10	1.52	1.74
Fibrinogen Level (mg/dL)	200-400	577	584
Others			
Ammonia (μmol/L)	18-72	40	810
Lactic Acid (mmol/L)	0.7-2.0	5.9	10.9

BUN, blood urea nitrogen; AST, aspartate aminotransferase; ALT, alanine aminotransferase; ALP, alkaline phosphatase; INR, international normalized ratio.

Six hours later, the patient developed bilateral upper and lower extremity myoclonic jerks. Epileptiform activities were absent on real-time video electoctroencephalogram. These new symptoms were attributed to the multi-segmental myoclonus of grade IV HE. A repeat head CT scan revealed diffuse partial cerebral sulcal effacement consistent with cerebral edema. The patient was deeply sedated with propofol and midazolam to achieve a goal of -4 to -5 on the Richmond Agitation-Sedation Scale (RASS). Permissive hypocapnia, therapeutic hypothermia, osmotic therapy with hypertonic saline and mannitol, and broad-spectrum antibiotics were immediately initiated. Lactulose, rifaximin, zinc sulfate, and continuous veno-venous hemodialysis (CVVHD) were started to attempt to decrease ammonia levels. High volume TPE was performed concurrently with CVVHD over the next two days.

**Figure 1:**
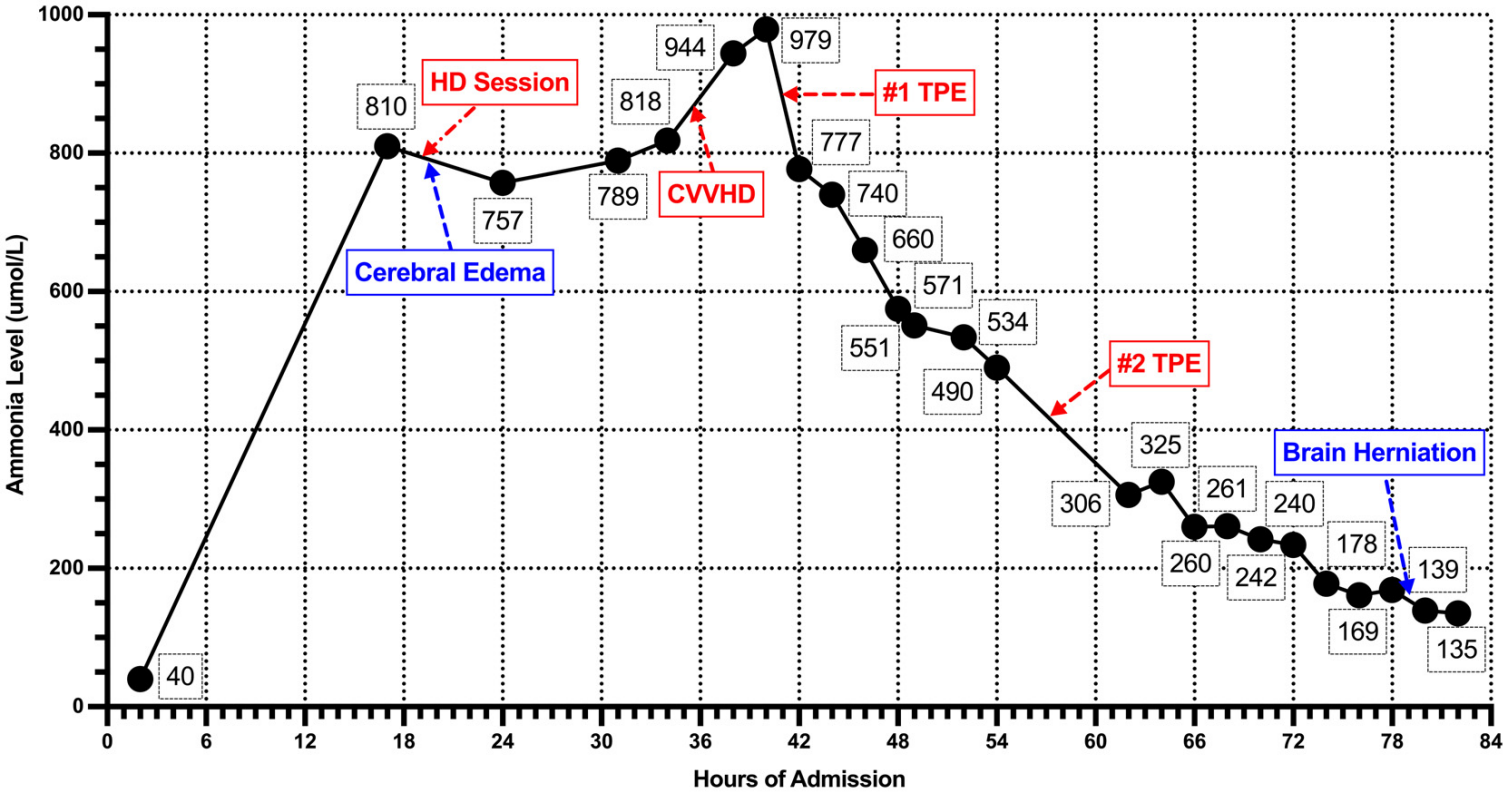
Rapid neurological deterioration despite continuous reduction in serum ammonia level. HD, hemodialysis; CVVHD, continuous veno-venous hemodialysis; TPE, therapeutic plasma exchange; #1, the first session; #2, the second session.

Despite intensive treatment and reduction of the ammonia level from a maximum value of 979 to 169 µmol/L (**Figure 1**), the patient’s condition continued to deteriorate. Repeat head CT 72 hours after admission showed worsening cerebral and cerebellar edema with new bilateral uncal herniation, downward tonsillar/central herniation and associated pontine Duret microhemorrhages. Twelve hours later, the patient was declared brain dead.

## Discussion

HE is commonly seen in patients with ACLF. Data from the CANONIC trial suggested that HE occurred more frequently in patients with the following: younger age, alcohol-associated liver disease, active alcohol abuse, severe liver failure with a systemic inflammatory reaction, bacterial infection, or dilutional hyponatremia (4, 5).

Cerebral edema is a cardinal feature of ALF. It occurs in approximately 75% of ALF patients with Grade IV HE and 25-35% of patients with Grade III HE (6). Cerebral edema remains a leading cause of death among patients with ALF. However, HE complicated by cerebral edema occurs uncommonly in patients with cirrhosis and ACLF (7, 8). In a retrospective cohort of 81 patients with ACLF and high-grade HE, only three (3.7%) had clinically relevant cerebral edema (9).

Traditional risk factors of developing cerebral edema in ALF include a higher ammonia level at time of presentation, a higher grade of HE at time of admission, and a more acute clinical presentation (9).

Ammonia plays a central role in the pathophysiology of HE, as described in the “osmotic gliopathy theory” (10). Excess ammonia is converted into osmotically active glutamine in astrocytes. In patients with ALF, the most severe neurologic sequelae - including cerebral edema, seizures and brain herniation – typically occur when the serum ammonia acutely rises to a level greater than 200 μmol/L. Other potential drivers of cerebral edema in liver disease include: oxidative and nitrosative stress, proinflammatory cytokines, ion and water channel dysregulation, and neuronal lactate accumulation (1).

Ammonia levels correlate with the degree of HE in patients with CLD, but the relationship is not necessarily linear or exponential (11). In patients with ACLF, baseline arterial ammonia level is reported to be an independent predictor of 30-day mortality (12).

Comprehensive guidelines have been established for the management of ICH in ALF (3, 13, 14). However, no formal guidelines are available for patients with ICH in ACLF.

The early diagnosis of ICH in ACLF can be challenging. Very low-grade cerebral edema can be missed if a CT of the head is used instead of T2-weighted sequences on MRI (15). A finding of T2-weighted diffuse white matter hyperintensities may be associated with worse outcomes (8). Once ICH is diagnosed, optic nerve ultrasound and transcranial Doppler can be used to monitor the ICP in both ALF and ACLF (16).

In patients with ALF and ICH, hyperosmolar therapy with mannitol bolus is recommended as first-line management to lower the ICP (3, 14). Hypertonic saline is also recommended (3, 14). In one randomized trial, treatment with hypertonic saline reduced the incidence of ICH to 3 out of 15 treated ALF patients, as opposed to 7 of 15 control patients (17).

Induction of a pharmacologic coma can be utilized as a bridge to liver transplantation (LT) when severe ICH fails to respond to other measures (18). Barbiturate-induced coma can minimize cerebral metabolic activity and reduce ICP. Moderate hypothermia (target core temperature of 33-34°C) may also be used as a bridge therapy to LT in cases of refractory ICH. A 2010 systematic review suggested a benefit of moderate hypothermia in control of ICP, cerebral perfusion pressure, and cerebral blood flow (19). However, a more recent randomized trial showed that moderate hypothermia did not prevent ICH or improve survival in patients with severe acute liver failure (20).

Lactulose and rifaximin are the mainstays of therapy for overt HE due to CLD. Ammonia-reducing therapy is commonly instituted in the management of ICH in both ALF and ACLF (6). The actual utility of lactulose and rifaximin in ALF remains unclear. There have been no studies on the utility of these medications in ACLF.

Early initiation of CRRT is effective at reducing ammonia and improving metabolic equilibrium and fluid balance in patients with ALF (21). CRRT lowers ammonia levels within three days of initiation and improves 21-day transplant-free all-cause mortality in patients with ALF (22). Furthermore, a 2018 retrospective analysis including patients with ACLF demonstrated that CRRT with concurrent hypertonic saline infusion improved cerebral edema and HE severity (23).

TPE has been added to the armamentarium of therapies as an evidence level I, grade 1 recommendation for the management of ALF (24). TPE is theorized to remove circulating toxins and inflammatory mediators, replace coagulation factors, and modulate the immune system (24). A randomized trial of TPE in 182 patients with ALF reported significant improvement in transplant-free survival compared to standard medical therapy (25). TPE was also trialed in patients with ACLF. A meta-analysis of four studies of TPE-based therapy revealed improved survival at 30 and 90 days in non-transplanted patients with ACLF as compared to standard medical therapy (26). Reduction in serum ammonia was consistently observed in ACLF patients undergoing treatment with TPE. However, these improvements did not correlate with survival outcome (26).

**Figure 2:**
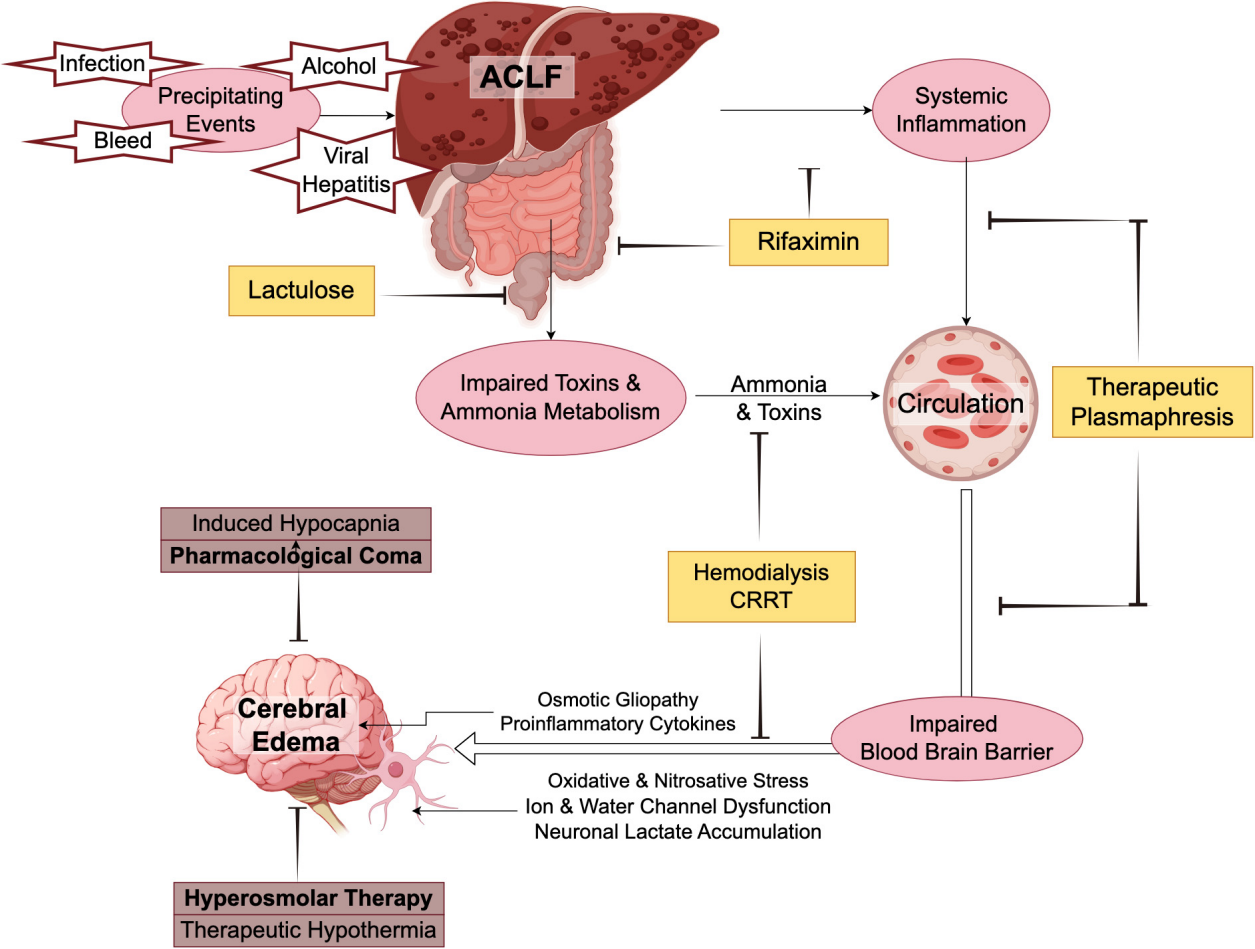
Pathophysiology and potential treatment approaches of cerebral edema in acute-on-chronic liver failure.

**Figure 2** summarizes the pathophysiology and potential treatment approaches for ICH in ACLF.

Our patient was treated with a combination of mannitol, hypertonic saline, lactulose, rifaximin, zinc sulfate, CVVHD and TPE, which collectively led to a rapid reduction in the patient’s serum ammonia. Unfortunately, the patient continued to experience neurological deterioration with worsening ICH and ultimate brain herniation. This suggests that factors other than ammonia may be contributing to ICH.

## Conclusion

ICH and cerebral edema are uncommon complications in patients with CLD and ACLF. When they arise, they demand aggressive therapy. Many of the treatment strategies used in managing patients with ALF and ICH have been adopted in patients with ACLF. Treatment options include ammonia-reducing drugs, ICP-lowering therapies (including mannitol and hypertonic saline), CRRT and TPE. Further studies are needed to clarify the risk factors for developing HE and cerebral edema in ACLF. Additionally, research should focus on validating various treatment approaches, including CRRT and TPE, to determine the appropriate patient population and the optimal treatment window for each approach.
